# Resistance training decreases plasma levels of adipokines in postmenopausal women

**DOI:** 10.1038/s41598-020-76901-w

**Published:** 2020-11-16

**Authors:** Liam J. Ward, Sigrid Nilsson, Mats Hammar, Lotta Lindh-Åstrand, Emilia Berin, Hanna Lindblom, Anna-Clara Spetz Holm, Marie Rubér, Wei Li

**Affiliations:** 1 Department of Obstetrics and Gynaecology in Linköping, and Department of Biomedical and Clinical Sciences, Linköping University, Linköping, Sweden; 2 Occupational and Environmental Medicine Center in Linköping, and Department of Health, Medicine and Caring Sciences, Linköping University, 58185 Linköping, Sweden; 3 Department of Clinical Science, Intervention and Technology, Karolinska Institutet, 14186 Stockholm, Sweden; 4 Department of Health, Medicine and Caring Sciences, Unit of Physiotherapy, Linköping University, Linköping, Sweden

**Keywords:** Lifestyle modification, Cardiovascular diseases, Randomized controlled trials

## Abstract

Physical inactivity and the onset of menopause increase the risk of cardiovascular disease amongst postmenopausal women. We aim to investigate the effect of resistance training (RT) on plasma levels of selected cytokines, adipokines, myokines, and sex hormones in postmenopausal women with vasomotor symptoms. This was a sub-study of a randomised controlled trial investigating the effects of RT on vasomotor symptoms in postmenopausal women. Women were randomised to join a 15-week RT program (n = 26) or remain sedentary as control (n = 29). Venous blood samples were taken at week-0 and week-15 for all participants. Enzyme-linked immunosorbent assays and multiple bead assays were used to measure cytokines, adipokines, myokines, and sex hormones in plasma. Plasma measurements of 16 of 33 analytes were within detectable limits. After adjusting for good compliance in the RT group (58% of RT participants), after 15 weeks, significantly lower plasma levels of adiponectin (p < 0.001), lipocalin-2 (p < 0.01) and resistin (p = 0.04) were found. Comparing control and RT women, using change-over-time values, significant increases in median testosterone and sex hormone binding globulin levels were seen in RT women. RT intervention lowers the levels of adipokines, particularly adiponectin, in postmenopausal women with vasomotor symptoms. These results were secondary outcomes of a clinical trial, and further investigations in a larger cohort are essential with the additional control of diet control and body composition analyses. Nevertheless, our study shows RT may be a beneficial intervention in reducing inflammation amongst postmenopausal women.

## Introduction

Women can now expect to live more than a third of their lives after menopause^1^. The incidence of cardiovascular disease (CVD) increases after menopause due to reduced energy expenditure, redistribution of body fat, changed lipoprotein metabolism, and increased systemic inflammation in females^2,3^. These changes are reflected in the increased risk of CVD that approximately doubles during the 10 years after menopause^4^. Reduced levels of oestrogens, and relative increases in androgens, have, aside from aging per se, been attributed to these physiological changes and increased CVD risk in postmenopausal women^5,6^.

Systemic inflammation is a risk factor for CVD^7^, and after menopause there is an increase in pro-inflammatory cytokines, including interleukin-1 (IL-1), IL-6 and tumour necrosis factor (TNF)^8^. Both IL-6 and the acute-phase protein C-reactive protein (CRP) have been identified as independent predictors of CVD events in postmenopausal women, with or without hormone-replacement therapy^9^. Obesity, body fat amount and fat distribution, are also risks for CVD that are inextricably linked with inflammation^10^.

Adipose tissue, as an endocrine organ, can secrete cytokines (referred to as adipokines) that exhibit both pro-inflammatory and anti-inflammatory effects^11^. Adiponectin, a key adipokine, has multiple biological functions on balancing glucose and lipid metabolism, insulin-sensitising, anti-apoptotic, anti- and pro-inflammatory, and oxidative stress^12^. It has been implicated in pathophysiology of several disorders, such as type 2 diabetes, metabolic syndrome, vascular disease, autoimmune diseases, osteoporosis, and cancer^12–15^. For women in midlife there is a tendency for weight gain and central fat distribution^16^, which can shift the balance of adipokine secretion to a more pro-inflammatory state. In postmenopausal women, plasma concentrations of adiponectin are increased^17^ and the levels of adiponectin negatively correlated with bone mineral density, serum oestradiol concentration and monocyte chemotactic protein-1^18,19^. However, in postmenopausal women lower levels of adiponectin are found in obese and overweight patients as compared with healthy weight women^20^. It should also be noted that varying isoforms of adiponectin, such as high molecular weight adiponectin, are known to exert different biological effects^21^.

Lack of physical exercise is a known risk factor for the development of CVD and all-cause mortality in postmenopausal women^22^. Regular exercise has shown to have numerous benefits amongst postmenopausal women, from weight loss, alterations in lipid profiles, to reductions in oxidative stress and inflammation^23,24^. Moderate aerobic exercise, and resistance training (RT) have both shown beneficial effect in lowering inflammatory activity in women^25^. Additionally, high-intensity interval training over 12 weeks (28 min of high intensity exercise with > 80% of maximum heart rate) reduced visceral adiposity tissue and increased IL-6 in obese postmenopausal women^26^, and increased resistance training volume enhanced muscle hypertrophy in postmenopausal women^27^. Recently, within this cohort, RT has been shown to reduce hot flush frequency by nearly half in those postmenopausal women^28^. Moreover, studies have demonstrated the beneficial effects of RT on postmenopausal women, such as in improvement of muscle strength, metabolic risks, inflammation, lipid profiles, mental health and decrease of ferritin^26,27,29–31^. The beneficial effects of RT on postmenopausal women may depend on training volume and time. It has been shown that 12-week RT improved physical fitness, mental health, quality of sleep and changes of visceral fat to skeletal muscle area ratio in postmenopausal women^30^.

The effect of exercise on the levels of adipokines both systemically and locally in adipose tissue is a growing area of study. However, due to difficulties in stratifying influencing factors there is still no consensus on the effect of exercise. Specifically in postmenopausal women without hormone-replacement therapy, observational studies show a negative associations^32^, whereas intervention studies remain inconclusive^33^.

The aim of this study was to determine if a 15-week resistance training (RT) regime of physical exercise can alter the plasma profile of CVD markers, inflammation markers, adipokines, and myokines in a cohort of postmenopausal women. The hypothesis of the study is that postmenopausal women in the RT group will have significantly reduced levels of proinflammatory analytes and/or increased levels of anti-inflammatory analytes after the 15 weeks study period.

## Material and methods

### Participants

Postmenopausal women with vasomotor symptoms (n = 65) were recruited to an open, parallel group, randomised controlled intervention study (clinical trial registered ID: NCT01987778) conducted at Linköping University Hospital, Sweden. The study was performed according to the Declaration of Helsinki and relevant parts of Good Clinical Practice. The research protocol was approved by the Regional Ethical Review Board in Linköping (2013/285-31 and 2013/338-32), and the trial design and protocol previously published^34^. Written informed consent was obtained from all participants prior entry to the trial. The primary outcome of the trial was to investigate if RT could be associated with a reduction in vasomotor symptoms.

Inclusion/exclusion criteria and details of the RT programme have previously been reported^34^. Briefly, inclusion criteria included; postmenopausal women, ≥ 45 years old, good general health with physical ability to participate in the RT regime. Exclusion criteria included; > 75 min per week of moderate- to vigorous-intensity physical activity, > 225 min per week of any physical activity, capillary haemoglobin < 110 g/L, blood pressure systolic > 160 mmHg or diastolic > 100 mmHg, systemic hormone therapy or other therapy possibly decreasing hot flushes (antidepressants were allowed if the dose was stable and treatment unrelated to vasomotor symptoms), and medical conditions limiting full participation in RT.

**Figure 1:**
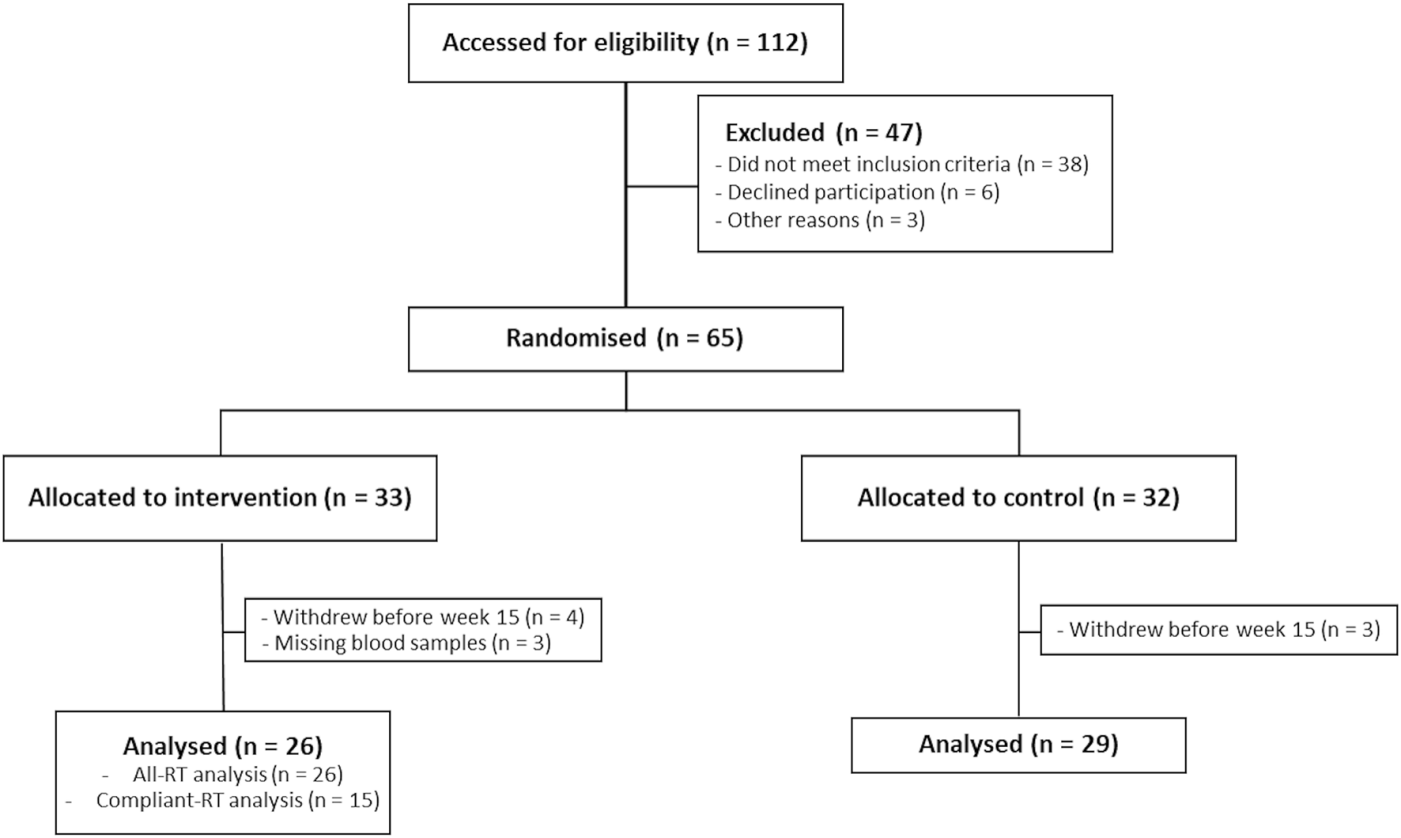
Study flow chart. Postmenopausal women were recruited and randomly allocated to the intervention (n = 33) or control group (n = 32). Two series of analyses were performed in the resistance training (RT) intervention group, all-RT and compliant-RT, based on the participants good compliance to the resistance training regime.

Participants were randomly assigned to either control (remain sedentary) or intervention (RT) groups for a study period of 15 weeks (Fig. 1). Participant allocation was performed using block randomisation through sequentially numbered sealed envelopes, only opened upon participant inclusion. Group allocation remained blinded to investigators performing analyses until the conclusion of the study.

### Intervention

The RT intervention comprised of 15 weeks structured RT performed three times per week targeting all major muscle groups. The regime consisted of six exercises in seated resistance machines and two body-weight exercises in the following order; chest press, leg press, seated row, leg curl, latissimus dorsi pull-down, leg extension, crunches and back raises. The seated exercises were performed in 15–20 repetitions in two sets week 1–3, and 8–12 repetitions in two sets week 4–15. Body-weight exercises were performed until exhaustion in two sets^28,34^. A physiotherapist supervised one session per week to assess fidelity to the training schedule, which was adapted to each individual with gradually increased loads during the trial. Strength tests using 8 repetition-maximum (8 RM) tests were put through in the seated resistance machines at baseline, three weeks, and 15 weeks in the intervention group. Any potential changes in the 8 RM tests between baseline and three weeks were believed to be mainly attributed to neuromuscular adaptations, whereas changes from three weeks to 15 weeks were believed to be attributed to muscle hypertrophy^35^.

Personal physical activity logs, together with logs from the electronic card system at the designated gym, were recorded to track compliance. Established study criteria stipulated good compliance as an average of two or more RT sessions per week, excluding time-off due to illness^34^. Participants in the control group were instructed to remain sedentary, and not change their level of physical activity for the study period.

A total of 58 women completed the study, 29 controls and 29 RT, with seven women being excluded due to fulfilling withdrawal criteria. In addition, another three women in the RT groups were excluded due to missing plasma samples from the two time-points, giving a total of 26 in RT group. Within the RT group, the number of participants with good compliance to the RT protocol was 15 out of 26 women (58%), whereas 11 women had an average of below two RT sessions per week.

### Clinical information, anthropometric measures, and plasma sample collection

Basic clinical information was recorded at baseline, including; age, blood pressure, haemoglobin, medication use, and smoking (Table 1). Anthropometrics were measured at both week-0 and week-15, including; weight and body mass index (BMI), and abdominal circumference and half-width.

**Table 1:** Baseline clinical information of study participants. There were no significant differences between groups in any of the variables.

	Control (n = 29)	All-RT (n = 26)
Age, years (SD)	55.4 (5.0)	55.7 (5.1)
Blood pressure, mmHg (SD)		
Systolic	128.3 (16.6)	131.1 (14.0)
Diastolic	78.4 (10.1)	78.5 (7.1)
Haemoglobin, g/L (SD)	140.4 (7.8)	137.1 (9.9)
Medication used for, n (%)		
Hypertension	3 (10)	7 (27)
Rheumatoid arthritis	1 (3)	1 (4)
Hypothyroidism	2 (7)	3 (12)
Crohn’s disease	1 (3)	1 (4)
Smoking, n (%)	1 (3)	1 (4)

*RT* resistance training, *SD* standard deviation.

Fasting blood samples, collected in EDTA vacutainers (BD AB, Sweden), were obtained at week-0 and again at week-15 of the study period for each participant. Plasma was isolated from the blood samples and after centrifugation at 1500×*g*, aliquoted and stored at − 70 °C until future analyses.

### Analyses of adipokines, cytokines, and myokines by multiplex bead array

The MILLIPLEX multiplex assays (Merck, Germany) using xMAP technology (Luminex Corporation, TX, USA) was used to analyse the levels of various analytes within the plasma samples. MILLIPLEX magnetic beads panels (Merck) were used to analyse 37 analytes across 11 bead panels, including: inflammatory cytokines and chemokines, adipokines, myokines, and other analytes. A full list of analytes can be found in Supplementary Table S1.

All procedures were performed according to the manufacturer guidelines. Standard curves were created for each analyte, using either a six- or seven-standard concentrations depending on manufacturer guidelines for each panel. Two quality controls provided in the kits were added to each panel. Analysis of Luminex panels was performed using the Luminex 200 (Luminex Corporation) instrument, for acquisition the xPONENT software (v.3.1.7; Luminex Corporation) was used, and data analysis was performed using the Masterplex 2010 software (v.2.0.0.68; Mirai Bio Group, CA, USA). The median fluorescent intensity was analysed using a 5-parameter logistic curve-fitting to calculate the concentration of analytes in each sample.

### Analysis of testosterone by enzyme-linked immunosorbent assay

Enzyme-linked immunosorbent assay (ELISA) was used for the measurement of testosterone (DE1559; Demeditec Diagnostics, Germany) in plasma samples. Procedures were carried out in accordance to the manufacturer guidelines. Plasma samples (25 µL) were loaded onto the plate in duplicate. Data were analysed using six standard concentrations and a 4-parameter logistic curve-fitting to calculate the concentration of testosterone in each sample. The average concentration from each set of duplicates was taken to give the final testosterone concentration, expressed as ng/mL (conversion to SI unit: nmol/L = ng/mL × 3.48).

### Statistics

Data are expressed as either mean ± standard deviation (SD) or median ± quartile range, dependent on data distribution, unless otherwise stated. Two series of statistical analyses were performed, based on: (1) treatment intention, where results from all RT participants who had measurements from the baseline to 15 weeks regardless of the level of compliance (herein termed: “*all-RT*”), and (2) the per-protocol principle, where results from only RT participants with good compliance (herein termed “*compliant-RT*”).

Data comparisons between week-0 and week-15 in either control or RT groups were performed using non-parametric Wilcoxon-signed rank statistics, unless otherwise stated. Comparisons between control and RT groups after 15 weeks were performed using non-parametric Mann–Whitney *U* test, by using a percentage of the week-15 values to the corresponding week-0 values (percentage change from baseline). In addition, generalised estimating equation (GEE) analysis was performed to assess the intervention by group and time interactions. All statistical analyses were performed in SPSS (v.25.0 & v.27.0, IBM, UK).

## Results

There were no significant differences between the control and RT groups in demographics and clinical information at the time of enrolment (Table 1). In addition, there were no differences in body anthropometrics, adipokines, myokines, cytokines, and other measured analytes at week-0 of the study period (Supplementary Table S2). Thus, clinical, anthropometric, and plasma analytes data indicate that the study randomisation was successful.

Body anthropometrics were measured for all women included in the study at week-0 and week-15 (Table 2). No significant differences were found in the recorded parameters over the study period for women in either control or RT groups.

**Table 2:** Body anthropometrics measured in each study group at 0-weeks and at 15-weeks of the study period.

	Control (n = 29)		*p*	All-RT (n = 26)		*p*	Compliant-RT (n = 15)		*p*
	Week-0	Week-15		Week-0	Week-15		Week-0	Week-15	
Weight (kg)	72.3 (11.5)	72.4 (11.9)	0.75	76.5 (11.5)	75.9 (11.6)	0.17	75.8 (9.9)	75.2 (10.0)	0.41
BMI	26.7 (3.6)	26.8 (3.8)	0.79	28.1 (3.8)	27.9 (3.9)	0.14	27.9 (2.9)	27.7 (3.0)	0.36
Abdominal (cm)									
Circumference	88.8 (12.9)	89.4 (14.1)	0.55	92.7 (11.4)	90.6 (10.2)	0.12	91.3 (7.4)	90.8 (8.5)	0.70
Half-width	17.0 (2.6)	17.2 (2.7)	0.44	17.6 (2.6)	17.5 (2.6)	0.59	17.7 (2.6)	17.9 (2.4)	0.56

Data expressed as mean (standard deviation).

Paired t-test was used to compare measured parameters across the 15-week study period.

*BMI* body mass index, *RT* resistance training.

In the 8 RM strength tests from baseline to three weeks strength increases of 13.7–33.4% were seen, and from three weeks to 15 weeks increases from 19.1 to 25.1% were seen, dependent on the tested muscle groups. These occurred without changes to BMI (Table 2).

The plasma measurements of 16 analytes were within detectable limits and concentrations are presented in Table 3. A significant difference was found in the control group, between week-0 and week-15, with increases in MCP-1 (p = 0.02). Significant differences in the all-RT group and the compliant-RT group, between week-0 and week-15, included decreases in adiponectin, lipocalin-2, and an increase in testosterone. In addition, in the compliant-RT group, a significant decrease in resistin (p = 0.04) was present. Among the non-complaint-RT women (those who dropped out), there were no significant differences in adiponectin, lipocalin-2, resistin, and testosterone (Supplementary Table S3).

**Table 3:** Plasma parameters measured at 0-weeks and at 15-weeks resistance training (RT).

	Control (n = 29)		*p*	All-RT (n = 26)		*p*	Compliant-RT (n = 15)		*p*
	Week-0	Week-15		Week-0	Week-15		Week-0	Week-15	
Adiponectin (µg/mL)^†^	40.6 (38.1)	34.1 (46.7)	0.24	34.3 (37.8)	27.9 (34.4)	< 0.001***	36.4 (45.1)	21.0 (35.2)	< 0.001***
Adipsin (µg/mL)	3.5 (0.9)	3.8 (0.78)	0.37	3.7 (0.7)	3.6 (0.9)	0.07	3.8 (0.9)	3.7 (0.9)	0.28
BDNF (ng/mL)^†^	1.4 (2.8)	1.4 (2.4)	0.96	2.1 (2.2)	1.5 (3.4)	0.52	1.4 (2.1)	1.5 (1.2)	0.54
CRP (µg/mL)	9.4 (16.4)	8.7 (15.5)	0.76	9.1 (12.4)	8.6 (12.1)	1.00	11.5 (13.4)	9.3 (12.4)	0.39
Leptin (ng/mL)^†^	26.2 (33.8)	25.4 (28.7)	0.61	31.7 (23.1)	29.2 (25.3)	0.17	32.2 (22.1)	31.5 (19.7)	0.36
Lipocalin-2 (µg/mL)	96.3 (52.9)	93.8 (33.1)	0.14	89.0 (25.0)	84.4 (20.5)	0.03*	95.2 (26.4)	84.3 (20.0)	< 0.01**
MCP-1 (pg/mL)	312.0 (115.5)	345.8 (117.1)	0.02*	302.5 (160.5)	292.7 (173.7)	0.22	279.5 (153.7)	262.5 (166.1)	0.21
MMP-2 (ng/mL)	127.2 (38.3)	118.9 (32.7)	0.66	118.6 (20.1)	113.1 (29.8)	0.36	126.3 (17.9)	123.9 (27.3)	0.25
MMP-9 (ng/mL)	39.7 (18.5)	35.5 (21.4)	0.24	30.4 (29.9)	28.4 (18.6)	0.36	38.2 (30.3)	26.8 (19.9)	0.11
Osteonectin (pg/mL)^†^	343.9 (242.0)	334.3 (242.2)	0.67	370.4 (164.0)	343.9 (168.3)	0.99	332.9 (186.9)	310.0 (130.7)	0.39
PAI-1 (ng/mL)	34.6 (19.9)	39.3 (32.1)	0.74	32.8 (16.6)	28.2 (28.1)	0.19	30.3 (10.7)	26.6 (20.6)	0.30
Resistin (ng/mL)	27.5 (13.4)	25.5 (14.4)	0.29	25.5 (9.8)	24.4 (10.9)	0.07	26.8 (9.7)	25.2 (10.1)	0.04*
SHBG (nM)	81.1 (42.5)	76.8 (45.8)	0.41	86.0 (33.7)	88.9 (34.0)	0.14	72.8 (40.0)	84.5 (33.9)	0.08
Testosterone (ng/mL)	0.8 (0.3)	0.8 (0.3)	0.53	0.7 (0.2)	0.8 (0.2)	0.04*	0.8 (0.4)	0.8 (0.2)	0.05*
Testosterone/SHBG (%)	3.9 (3.5)	3.9 (3.8)	0.85	3.2 (2.3)	3.6 (2.1)	0.59	3.6 (1.8)	3.7 (2.1)	0.89
TNFα (pg/mL)	30.4 (31.8)	35.9 (31.9)	0.73	34.3 (28.1)	32.6 (34.3)	0.40	32.6 (27.11)	33.7 (18.6)	0.67

Data presented as Median (IQR = Q3–Q1). Significance *p ≤ 0.05, **p < 0.01, ***p < 0.001.

Wilcoxon-signed rank tests was used to compare measured parameters within each study group across the 15-week study period.

Testosterone unit conversion factor: nmol/L = ng/mL × 3.48.

*BDNF* brain derived neurotropic factor, *CRP-C* reactive protein, *MCP* monocyte chemoattractant protein, *MMP* matrix metalloproteinase, *PAI* plasminogen activator inhibitor, *SHBG* sex hormone binding globulin, *TNF* tumour necrosis factor.

^†^Different numbers due to limits-of-detection: *Adiponectin*—Control (n = 21), All-RT (n = 23), Compliant-RT (n = 14); *BDNF*—Control (n = 26), All-RT (n = 25), Compliant-RT (n = 14); *Leptin*—Control (n = 27); *Osteonectin*—Control (n = 26), All-RT (n = 25), Compliant-RT (n = 14).

**Figure 2:**
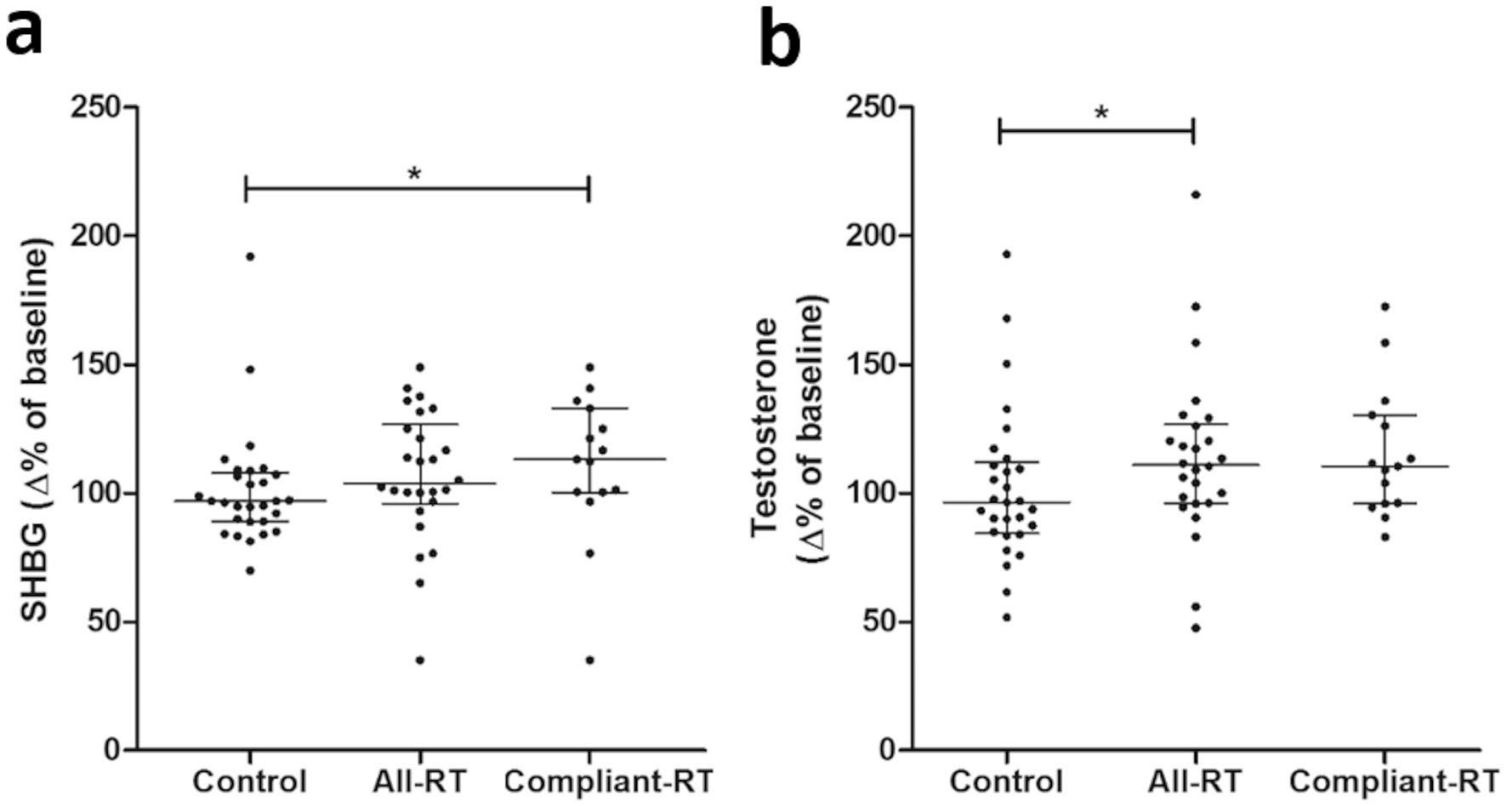
The effect of 15-weeks resistance training (RT) on sex hormone binding globulin (SHBG) and testosterone in postmenopausal women. Postmenopausal women were randomised into either control (n = 29) or RT groups, presented as both all-RT (n = 26) and compliant-RT (n = 15). Plasma levels of (A) SHBG and (B) testosterone were measured at week-0 and week-15 of the study period. Values were normalised to baselines values (% of corresponding week-0 values).

Median increases in sex hormone binding globulin (SHBG) and testosterone levels were observed in both all-RT and compliant-RT groups, which is consistent with a previous study^30^. No significant differences were observed when comparing the testosterone/SHBG fraction, a proxy for bioavailable testosterone, in any group (Table 3). Significant differences between groups were assessed with values representing change-over-time, using week-15 values normalised with corresponding week-0 value (percent change from baseline). Significant differences between groups were found in SHBG change-over-time between control and compliant-RT groups (Fig. 2A), and testosterone change-over-time between control and all-RT groups (Fig. 2B).

To assess the potential causal relationship, generalised estimating equation (GEE) was performed. We found that testosterone (p = 0.05) and adiponectin (p = 0.002) showed significant interactions, in the all-RT and compliant-RT models, respectively (Table 4). Trends were also observed for adiponectin and adipsin, in the all-RT model, and testosterone in the compliant-RT model (Table 4).

**Table 4:** Generalised estimating equations (GEE) results for the outcome of plasma parameters.

	All-RT^†^			Compliant-RT^††^		
	Group *p*	Time *p*	Group × time *p*	Group *p*	Time *p*	Group × time *p p*
Adiponectin	0.54	0.68	**0.089**^**#**^	0.34	0.42	0.002**
Adipsin	0.55	0.91	**0.094**^**#**^	0.37	0.81	0.11
BDNF	0.74	0.42	0.34	0.27	0.73	0.86
CRP	0.41	0.88	0.97	0.33	0.85	0.70
Leptin	0.35	0.14	0.93	0.47	0.13	0.78
Lipocalin-2	0.06^#^	0.04*	0.61	0.07^#^	0.001**	0.47
MCP-1	0.40	0.01*	0.32	0.08^#^	0.01*	0.35
MMP-2	0.74	0.33	0.97	0.23	0.22	0.81
MMP-9	0.75	0.16	0.73	0.84	0.12	0.41
Osteonectin	0.83	0.58	0.28	0.28	0.25	0.68
PAI-1	0.71	0.52	0.53	0.10	0.66	0.46
Resistin	0.67	0.23	0.42	0.93	0.08^#^	0.16
SHBG	0.67	0.41	0.60	0.97	0.19	0.30
Testosterone	0.40	0.16	0.050*	0.73	0.19	**0.058**^**#**^
Testosterone/SHBG	0.17	0.31	0.56	0.46	0.45	0.64
TNFα	0.63	0.80	0.69	0.33	0.54	0.64

Significance *p ≤ 0.05, **p < 0.01; Trending ^**#**^*p* < 0.10.

^**†**^All-RT includes all participants irrespective of reaching the intervention compliance threshold.

^**††**^Compliant-RT includes only participants that completed, on average, two or more exercise session per week.

Group: control and RT, Time: Baseline and 15 weeks.

*BDNF* brain derived neurotropic factor, *CRP* C-reactive protein, *MCP* monocyte chemoattractant protein, *MMP* matrix metalloproteinase, *PAI* plasminogen activator inhibitor, *SHBG* sex hormone binding globulin, *TNF* tumour necrosis factor.

## Discussion

Physical activity is one of the most effective behavioural strategies to maintain and improve overall cardiometabolic health in the population. Physical inactivity together with the onset of menopause increases the risk of CVD amongst postmenopausal women. Therefore, the early introduction of regular training could be beneficial for women who are in the risk group for cardiometabolic disturbances associated with their menopausal transition, especially for women with vasomotor symptoms^36,37^.

In the present study, over a 15-week study period, we observe significant decreases in the adipokines; adiponectin, lipocalin-2, and resistin in the RT groups, with the former two being significant in both the all-RT and the compliant-RT groups. Additionally, testosterone levels were significantly altered in the RT groups when comparing week-15 and week-0 levels. Moreover, RT women had greater increases in testosterone and SHBG plasma levels when compared to the control group.

Lipocalin-2 and resistin are both pro-inflammatory adipokines and have been associated with CVD mechanisms. Lipocalin-2, also known as neutrophil gelatinase-associated lipocalin (NGAL), is mainly produced in adipocytes in white adipose tissue, and modulates the inflammatory response via possession of NFκB binding sites^38^. Several studies relate lipocalin-2 to inflammatory and dysmetabolic conditions, including obesity, the metabolic syndrome and CVD^39–41^. In humans, resistin is mainly produced by peripheral blood mononuclear cells and macrophages, and repeated studies have shown that resistin, independently of CRP, predicts coronary artery disease^42,43^. Resistin has been shown to stimulate the formation of foam cells and to disturb normal endothelial function, which are risks for atherosclerosis and CVD development^44,45^. The potential for these adipokines to be more sensitive biomarkers for early CVD, compared to CRP, highlights the importance to reduce their levels in circulation^40^, especially in postmenopausal women whom have an increased risk of CVD. The results of this study are in line with other conducted studies investigating the effect of RT on adipokines in postmenopausal women, but to our knowledge is the first to demonstrate that 15-weeks of controlled RT decreases the level of lipocalin-2 in plasma. Similarly, reduced plasma levels of resistin have been observed with periodical-RT regimes in elderly postmenopausal women^33^.

Adiponectin is an anti-inflammatory adipokine, produced by both white and brown adipose tissue^46^. Adiponectin circulates in varying isoforms, including high-molecular weight, that can exert different biological effects^21^. The intervention led to alterations in adiponectin levels in compliant-RT women, and trending with all-RT women. This indicates that good compliance to the RT intervention has a critical effect on adiponectin levels. In the current study a significant decrease in plasma adiponectin levels was observed in RT women, which is in agreement with another study utilising RT intervention, with a weight-loss diet, in obese postmenopausal women^47^. The type of exercise may influence the effect on adipokine levels, as increases in plasma adiponectin levels have been observed in postmenopausal women participating in aerobic exercise^48^. Although adiponectin is an anti-inflammatory adipokine, and high levels could be perceived as beneficial, there is some debate whether high levels are physiologically beneficial. High levels of adiponectin in high-risk CVD populations have been associated with increased CVD mortality^49,50^. Further analyses of specific adiponectin isoforms, such as high-molecular-weight adiponectin, and/or expression of adiponectin receptors may help discern the mechanisms responsible for this observed decrease.

In accordance with both Phillips et al.^51^ and Balducci et al.^52^, the present reductions in adipokines can occur even without marked body composition changes. This may imply an independent anti-inflammatory effect of RT on adipokine production, although additional studies are required with the inclusion of both systemic plasma measurements and local adipose tissue measurements.

For postmenopausal women, the changes in sex hormones challenges previous results, presenting lowering effects of physical activity on free sex hormone levels, especially free testosterone^53^. Physical activity directly reduces sex hormone levels and increases the SHBG production mainly via a fat loss mediating mechanism^54,55^ and there is less consensus if it also can exert independent effects. This discrepancy in results may be caused by the different type, load, and intensity of the investigated exercise interventions. However, the present absolute changes in testosterone and SHBG are small and may lack clinical significance. In addition, the bioavailable testosterone fraction is not significantly altered in any group, due to the simultaneous increases in testosterone and SHBG.

Limitations must be taken into consideration with the current study. Firstly, the participant numbers are relatively low for each study group, which statistically increases error and decreases power. This study was one part of a trial investigating the effect of the RT on vasomotor symptoms in postmenopausal women, and the sample size was calculated from the trial’s primary outcome of reducing hot flush frequency. Moreover, although weekly supervised RT was employed, to stimulate compliance and fidelity to the training schedule, the compliance rate of RT women was low. This low compliance can only be speculated on, but the fact that most participants worked fulltime and practical challenges to participate in RT may have contributed. Additional variables were also not accounted for that may have effects on the outcomes of the intervention, particularly, alterations to fat and muscle mass. Whilst specific measures of fat and muscle mass were not currently available, we did observe strength increases in the 8 RM strength tests between weeks three and 15, without an increase in BMI. Diet could also have had an impact on our current results, although participants were advised not to alter their normal diets, the implementation of food diaries should be considered in future investigations. Complementary statistical analyses were performed with the exclusion of participants with inherently inflammatory conditions such as rheumatoid arthritis and Crohn’s diseases (Table 1), however this did not alter the main result. Medication use was recorded for all participants and was not matched between study groups due to the group randomisation.

## Conclusions

The findings indicate that supervised RT over a 15-week period, with good compliance, significantly lowers the levels of pro-inflammatory adipokines lipocalin-2 and resistin in postmenopausal women. However, significant reductions in anti-inflammatory adiponectin were also observed. In conclusion, our results may indicate the potential benefit to RT training in postmenopausal women for reducing the risk of adipokine-induced inflammation, although further studies are required with attention on circulating adiponectin isoforms. A key limitation in this study is the lack of body composition analyses, thus, whether the effects of RT protocol on blood markers occurred due to exercise, per se, or due to body fat reductions remains to be determined by further analyses of MRI data as a continuation of the research project.

## Supplementary information

Supplementary Information.

## References

[1] Lobo RA (2014). Prevention of diseases after menopause. Climacteric.

[2] Ozbey N, Sencer E, Molvalilar S, Orhan Y (2002). Body fat distribution and cardiovascular disease risk factors in pre- and postmenopausal obese women with similar BMI. Endoc. J..

[3] Lopez-Candales A, Hernández Burgos PM, Hernandez-Suarez DF, Harris D (2017). Linking chronic inflammation with cardiovascular disease: from normal aging to the metabolic syndrome. J. Nat. Sci..

[4] Lisabeth L, Bushnell C (2012). Menopause and stroke: an epidemiologic review. Lancet Neurol..

[5] Stice JP, Lee JS, Pechenino AS, Knowlton AA (2009). Estrogen, aging and the cardiovascular system. Future Cardiol..

[6] Lizcano F, Guzmán G (2014). Estrogen deficiency and the origin of obesity during menopause. BioMed. Res. Int..

[7] Pearson TA (2003). Markers of inflammation and cardiovascular disease. Circulation.

[8] Gameiro CM, Romão F, Castelo-Branco C (2010). Menopause and aging: changes in the immune system—a review. Maturitas.

[9] Pradhan AD, Manson JE, Rossouw JE (2002). Inflammatory biomarkers, hormone replacement therapy, and incident coronary heart disease: prospective analysis from the women's health initiative observational study. JAMA.

[10] Perry CD (2008). Centrally located body fat is related to inflammatory markers in healthy postmenopausal women. Menopause.

[11] Nakamura K, Fuster JJ, Walsh K (2014). Adipokines: a link between obesity and cardiovascular disease. J. Cardiol..

[12] Polito R (2020). The important role of adiponectin and orexin-A, two key proteins improving healthy status: focus on physical activity. Front. Physiol..

[13] Choi HM, Doss HM, Kim KS (2020). Multifaceted physiological roles of adiponectin in inflammation and diseases. Int. J. Mol. Sci..

[14] Toussirot E, Binda D, Gueugnon C, Dumoulin G (2012). Adiponectin in autoimmune diseases. Curr. Med. Chem..

[15] Hebbard L, Ranscht B (2014). Multifaceted roles of adiponectin in cancer. Best Prac. Res. Clin. Endocrinol. Metab..

[16] Kapoor E, Collazo-Clavell ML, Faubion SS (2017). Weight gain in women at midlife: a concise review of the pathophysiology and strategies for management. Mayo Clin. Proc..

[17] Jürimäe J, Jürimäe T (2007). Plasma adiponectin concentration in healthy pre- and postmenopausal women: relationship with body composition, bone mineral, and metabolic variables. J. Physiol. Endocrinol. Metab..

[18] Azizeh FY (2019). Circulatory pattern of cytokines, adipokines and bone markers in postmenopausal women with low BMD. J. Inflamm. Res..

[19] Miyatani Y (2008). Associations of circulating adiponectin with estradiol and monocyte chemotactic protein-1 in postmenopausal women. Menopause.

[20] Tisato V (2017). Low circulating TRAIL levels are associated with increase of resistin and lipocalin-2/ngal adipokines in postmenopausal women. Mediat. Inflamm..

[21] Fisman EZ, Tenenbaum AJCD (2014). Adiponectin: a manifold therapeutic target for metabolic syndrome, diabetes, and coronary disease?. Br. J. Sports Med..

[22] Oguma Y, Sesso H, Paffenbarger R, Lee I (2002). Physical activity and all cause mortality in women: a review of the evidence. Br. J. Sports Med..

[23] Bartfay W, Bartfay E (2013). A case-control study examining the effects of active versus sedentary lifestyles on measures of body iron burden and oxidative stress in postmenopausal women. Biol. Res. Nurs..

[24] Jarrete AP (2014). Influence of aerobic exercise training on cardiovascular and endocrine-inflammatory biomarkers in hypertensive postmenopausal women. J. Clin. Transl. Endocrinol..

[25] Soares FHR, de Sousa MBC (2013). Different types of physical activity on inflammatory biomarkers in women with or without metabolic disorders: a systematic review. Women Health.

[26] Nunes PRP (2019). Effect of high-intensity interval training on body composition and inflammatory markers in obese postmenopausal women: a randomized controlled trial. AGE.

[27] de Oliveira-Júnior GN (2020). Resistance training volume enhances muscle hypertrophy, but not strength in postmenopausal women: a randomized controlled trial. J. Strength Cond. Res..

[28] Berin E (2019). Resistance training for hot flushes in postmenopausal women: a randomised controlled trial. Maturitas.

[29] Nunes PRP (2016). Effect of resistance training on muscular strength and indicators of abdominal adiposity, metabolic risk, and inflammation in postmenopausal women: controlled and randomized clinical trial of efficacy of training volume. AGE.

[30] Yoon J-R, Ha G-C, Kang S-J, Ko K-J (2019). Effects of 12-week resistance exercise and interval training on the skeletal muscle area, physical fitness, and mental health in old women. J. Exerc. Rehabil..

[31] Ward LJ (2020). Does resistance training have an effect on levels of ferritin and atherogenic lipids in postmenopausal women? A pilot trial. Sci. Rep..

[32] Wu SH (2014). Nonexercise physical activity and inflammatory and oxidative stress markers in women. J. Womens Health.

[33] Botero JP (2013). Effects of long-term periodized resistance training on body composition, leptin, resistin and muscle strength in elderly post-menopausal women. J. Sports Med. Phys. Fitness.

[34] Berin E, Hammar ML, Lindblom H, Lindh-Åstrand L, Spetz Holm A-C (2016). Resistance training for hot flushes in postmenopausal women: randomized controlled trial protocol. Maturitas.

[35] Bird SP, Tarpenning KM, Marino FE (2005). Designing resistance training programmes to enhance muscular fitness: a review of the acute programme variables. Sports Med..

[36] Gray KE (2017). Vasomotor symptom characteristics: are they risk factors for incident diabetes?. Menopause.

[37] Muka T (2016). Association of vasomotor and other menopausal symptoms with risk of cardiovascular disease: a systematic review and meta-analysis. PLoS ONE.

[38] Abella V (2015). The potential of lipocalin-2/NGAL as biomarker for inflammatory and metabolic diseases. Biomarkers.

[39] Ni J (2013). Serum lipocalin-2 levels positively correlate with coronary artery disease and metabolic syndrome. Cardiovasc. Diabetol..

[40] Wu G (2014). Elevated circulating lipocalin-2 levels independently predict incident cardiovascular events in men in a population-based cohort. Arterioscler. Thromb. Vasc. Biol..

[41] Xiao Y (2013). Circulating lipocalin-2 and retinol-binding protein 4 are associated with intima-media thickness and subclinical atherosclerosis in patients with type 2 diabetes. PLoS ONE.

[42] Yaturu S, Daberry RP, Rains J, Jain S (2006). Resistin and adiponectin levels in subjects with coronary artery disease and type 2 diabetes. Cytokine.

[43] Reilly MP (2005). Resistin is an inflammatory marker of atherosclerosis in humans. Circulation.

[44] Lee TS (2009). Resistin increases lipid accumulation by affecting class A scavenger receptor, CD36 and ATP-binding cassette transporter-A1 in macrophages. Life Sci..

[45] Kawanami D (2004). Direct reciprocal effects of resistin and adiponectin on vascular endothelial cells: a new insight into adipocytokine-endothelial cell interactions. Biochem Biophys. Res. Commun..

[46] Iacobellis G (2003). echocardiographic epicardial adipose tissue is related to anthropometric and clinical parameters of metabolic syndrome: a new indicator of cardiovascular risk. J. Clin. Endocrinol. Metab..

[47] Ibáñez J (2010). Resistance training improves cardiovascular risk factors in obese women despite a significative decrease in serum adiponectin levels. Obesity.

[48] Wang X, You T, Murphy K, Lyles MF, Nicklas BJ (2015). Addition of exercise increases plasma adiponectin and release from adipose tissue. Med. Sci. Sports Exerc..

[49] Dekker JM (2008). Prognostic value of adiponectin for cardiovascular disease and mortality. J. Clin. Endocrinol. Metab..

[50] Teoh H, Strauss MH, Szmitko PE, Verma S (2006). Adiponectin and myocardial infarction: a paradox or a paradigm? The opinions expressed in this article are not necessarily those of the Editors of the European Heart Journal or of the European Society of Cardiology. Eur. Heart J..

[51] Phillips MD (2012). Resistance training reduces subclinical inflammation in obese, postmenopausal women. Med. Sci. Sports Exerc..

[52] Balducci S (2010). Anti-inflammatory effect of exercise training in subjects with type 2 diabetes and the metabolic syndrome is dependent on exercise modalities and independent of weight loss. Nutr. Metab. Cardiovasc. Dis..

[53] Ennour-Idrissi K, Maunsell E, Diorio C (2015). Effect of physical activity on sex hormones in women: a systematic review and meta-analysis of randomized controlled trials. Breast Cancer Res..

[54] van Gemert WA (2015). Effect of weight loss, with or without exercise, on body composition and sex hormones in postmenopausal women: the SHAPE-2 trial. Breast Cancer Res..

[55] de Roon M (2018). Effect of exercise and/or reduced calorie dietary interventions on breast cancer-related endogenous sex hormones in healthy postmenopausal women. Breast Cancer Res..

