# Supplementary material for: Resistance training decreases plasma levels of adipokines in postmenopausal women
Source: Sci Rep. 2020 Nov 16;10:19837. doi: 10.1038/s41598-020-76901-w (PMC7669850; doi:10.1038/s41598-020-76901-w)
Supplement: Supplementary file 1 — Supplementary Information. [file 41598_2020_76901_MOESM1_ESM.docx]

**SUPPLEMENTARY INFORMATION**

**Resistance training decreases plasma levels of adipokines in postmenopausal women**

Liam J. Ward^1,2,3^*, Sigrid Nilsson^1^, Mats Hammar^1^, Lotta Lindh-Åstrand^1^, Emilia Berin^1^, Hanna Lindblom^4^, Anna-Clara Spetz Holm^1^, Marie Rubér^1^, Wei Li^1^*

**List of Supplemental Information**

**Supplementary Table S1.** Complete list of analytes measured in plasma samples and corresponding Milliplex Bead Panel catalogue number.

**Supplementary Table S2.** Baseline (week-0) comparisons for body anthropometrics, adipokines, myokines, cytokines, and other measured analytes. Mann-Whitney *U* tests were used to compare control and all-RT values, and control and compliant-RT values.

**Supplementary Table S3.** Plasma parameters measured at 0-weeks and at 15-weeks resistance training (RT) for those participants that did not surpass the compliance threshold (non-compliant-RT women). Wilcoxon-signed rank tests was used to compare measured parameters across the 15-week study period.

**Supplementary Table S1.** Complete list of analytes measured in plasma samples and corresponding Milliplex Bead Panel catalogue number.

| **Milliplex Bead Panel catalogue number** | **Analytes** |
| --- | --- |
| HADK1MAP-61K | Adiponectin, Adipsin, Lipocalin-2, PAI-1, Resistin |
| HMYOMAG-56K | Fractalkine, Osteocrin, Irisin, Mysotatin, Apelin, FGF-21, LIF, Osteonectin, Oncostatin M, BDNF |
| HCYTOMAG-60K | IL-1RA, IL-4, IL-6, IL-7, IL-8, IL-10, IL-15, MCP1. TNFα |
| HSTCMAG-28SK | IL-1b, IL-4, IL-5, IL-6, IL-10, IL-12p70, IFNγ, TNFα |
| HCVD3MAG-67K | CRP |
| HCCBP3MAG-58 | SHBG |
| HMMP2MAG-55K | MMP-2, MMP-9 |
| HAGP1MAG-12K | Follistatin, Leptin |
| TGFBMAG-64K-03 | TGFβ1, TGFβ2, TGFβ3 |
| BDNF – brain-derived neurotrophic factor; CRP – C-reactive protein; FGF – fibroblast growth factor; IFN – interferon; IL – interleukin; LIF – leukaemia inhibitory factor; MCP – monocyte chemoattractant protein; MMP – matrix metalloproteinase; PAI – plasminogen activator inhibitor; SHBG – sex hormone binding globulin; TGF – transforming growth factor; TNF – tumour necrosis factor. | |

**Supplementary Table S2.** Baseline (week-0) comparisons for body anthropometrics, adipokines, myokines, cytokines, and other measured analytes. Mann-Whitney *U* tests were used to compare control and all-RT values, and control and compliant-RT values.

|  | **Control (n = 29)** | **All-RT (n = 26)** | **P-value** |
| --- | --- | --- | --- |
| *Weight (kg) | 72.3 (11.5) | 76.5 (11.5) | 0.16 |
| *BMI | 26.7 (3.6) | 28.1 (3.8) | 0.23 |
| *Abdominal (cm) |  |  |  |
| - Circumference | 88.8 (12.9) | 92.7 (11.4) | 0.20 |
| - Half-width | 17.0 (2.6) | 17.6 (2.6) | 0.55 |
| ^†^Adiponectin (µg/mL) | 40.6 (38.1) | 34.3 (37.8) | 0.59 |
| Adipsin (µg/mL) | 3.5 (0.9) | 3.7 (0.7) | 0.18 |
| ^†^BDNF (ng/mL) | 1.4 (2.8) | 2.1 (2.2) | 0.51 |
| CRP (µg/mL) | 9.4 (16.4) | 9.1 (12.4) | 0.96 |
| ^†^Leptin (ng/mL) | 26.2 (33.8) | 31.7 (23.1) | 0.25 |
| Lipocalin-2 (µg/mL) | 96.3 (52.9) | 89.0 (25.0) | 0.35 |
| MCP-1 (pg/mL) | 312.0 (115.5) | 302.5 (160.5) | 0.75 |
| MMP-2 (ng/mL) | 127.2 (38.3) | 118.6 (20.1) | 0.59 |
| MMP-9 (ng/mL) | 39.7 (18.5) | 30.4 (29.9) | 0.20 |
| Oncostatin M (pg/mL) | 13.1 (15.8) | 6.5 (11.0) | 0.52 |
| ^†^Osteonectin (pg/mL) | 343.9 (242.0) | 370.4 (164.0) | 0.69 |
| PAI-1 (ng/mL) | 34.6 (19.9) | 32.8 (16.6) | 0.96 |
| Resistin (ng/mL) | 27.5 (13.4) | 25.5 (9.8) | 0.82 |
| SHBG (nM) | 81.1 (42.5) | 86.0 (33.7) | 0.55 |
| Testosterone (ng/mL) | 0.8 (0.3) | 0.7 (0.2) | 0.09 |
| TNFα (pg/mL) | 30.4 (31.8) | 34.3 (28.1) | 0.99 |
| Data presented as Median (IQR = Q3 – Q1).  *Data are mean (SD)  ^†^ Different numbers due to limits-of-detection: *Adiponectin* - Control (n = 21), All-RT (n = 23); *BDNF* – Control (n = 26), All-RT (n = 25); *Leptin* – Control (n = 27); *Osteonectin* – Control (n = 26), All-RT (n = 25).  BDNF – brain derived neurotropic factor; BMI – body mass index; CRP- C-reactive protein; MCP – monocyte chemoattractant protein; MMP – matrix metalloproteinase; PAI – plasminogen activator inhibitor; SHBG – sex hormone binding globulin; TNF – tumour necrosis factor | | | |

**Supplementary Table S3.** Plasma parameters measured at 0-weeks and at 15-weeks resistance training (RT) for those participants that did not surpass the compliance threshold (non-compliant-RT women). Wilcoxon-signed rank tests was used to compare measured parameters across the 15-week study period.

|  | **Non-compliant-RT (n = 11)** | | ***P*-value** |
| --- | --- | --- | --- |
|  | **Week-0** | **Week-15** |  |
| ^†^Adiponectin (µg/mL) | 34.3 (47.7) | 31.7 (36.1) | 0.83 |
| Adipsin (µg/mL) | 3.6 (0.5) | 3.5 (1.2) | 0.65 |
| BDNF (ng/mL) | 2.3 (3.0) | 4.1 (7.9) | 0.82 |
| CRP (µg/mL) | 5.6 (14.8) | 7.0 (12.6) | 0.87 |
| Leptin (ng/mL) | 28.3 (25.4) | 21.7 (42.1) | 0.53 |
| Lipocalin-2 (µg/mL) | 85.8 (17.5) | 84.4 (34.2) | 0.58 |
| MCP-1 (pg/mL) | 354.8 (222.0) | 363.0 (243.6) | 0.72 |
| MMP-2 (ng/mL) | 111.1 (16.5) | 101.6 (21.5) | 0.67 |
| MMP-9 (ng/mL) | 29.1 (18.0) | 30.6 (18.7) | 0.41 |
| Osteonectin (pg/mL) | 414.0 (101.0) | 363.6 (327.0) | 0.45 |
| PAI-1 (ng/mL) | 39.1 (9.9) | 29.0 (29.2) | 0.77 |
| Resistin (ng/mL) | 23.7 (6.4) | 22.7 (15.9) | 0.82 |
| SHBG (nM) | 92.7 (43.0) | 93.7 (43.1) | 0.77 |
| Testosterone (ng/mL) | 0.7 (0.2) | 0.6 (0.3) | 0.14 |
| TNFα (pg/mL) | 36.9 (42.2) | 19.2 (47.5) | 0.31 |
| Data presented as Median (IQR = Q3 – Q1).  ^†^ Different numbers due to limits-of-detection: *Adiponectin* (n = 10)  BDNF – brain derived neurotropic factor; CRP- C-reactive protein; MCP – monocyte chemoattractant protein; MMP – matrix metalloproteinase; PAI – plasminogen activator inhibitor; SHBG – sex hormone binding globulin; TNF – tumour necrosis factor | | | |
